# Pitfalls with the MAKO Robotic-Arm-Assisted Total Knee Arthroplasty

**DOI:** 10.3390/medicina60020262

**Published:** 2024-02-02

**Authors:** Konstantinos Dretakis, Christos Koutserimpas

**Affiliations:** 1 2nd Department of Orthopaedics, “Hygeia” General Hospital of Athens, 15123 Athens, Greece; 2 Department of Orthopaedics and Traumatology, “251” Hellenic Air Force General Hospital of Athens, Kanellopoulou Av 3, 11525 Athens, Greece; 3 Department of Anatomy, School of Medicine, Faculty of Health Sciences, National and Kapodistrian University of Athens, 11527 Athens, Greece

**Keywords:** robotic surgery, knee surgery, passive navigation, complications joint surgery, future perspectives robotics, robotics in orthopaedics

## Abstract

Robotic-arm-assisted total knee arthroplasty (RATKA) with the MAKO system minimizes deviations in implant alignment and yields superior precision in implant position compared to a manual total knee arthroplasty. In this comprehensive commentary, we present and categorize the limitations and pitfalls of the procedure and we also provide recommendations for avoiding each limitation. The main surgeon-related limitations include prolonged operation duration, loose insertion of the checkpoints and pins, wrong registration and mapping, and damage to soft tissues during bone cutting. The system-related issues include the interruptions of the saw-cutting due to vibrations, specifications for the operating room floor and power supply, the high cost of the system, as well as the cost of each operation due to the extra implants, inability to use the system with various prostheses, wireless connection interruptions between the system’s components, and hardware issues with the six joints of the robotic device. In order to circumvent the potential challenges in this surgical procedure, it is essential to possess sufficient experience and undergo comprehensive training. Maintaining continuous awareness of the additional implants throughout the entire operation and prioritizing the preservation of soft tissues are of paramount significance. A profound comprehension of the system and its inherent constraints can also prove to be pivotal in certain situations.

## 1. Introduction

Over the years, there have been remarkable advancements in arthroplasty techniques and instrumentation [1,2]. Progress in joint reconstruction surgery has been marked by the adoption of a range of techniques and technologies, such as minimally invasive surgical procedures, accelerated recovery protocols, enhanced preoperative, intraoperative, and postoperative management in order to minimize the necessity for blood transfusions, and remarkable advancements in navigation and robotic systems [1,3,4,5,6].

One outstanding illustration of the evolving technology in joint replacement surgery is the MAKO Robotic Arm Interactive Orthopaedic System (Mako Surgical Corporation, Kalamazoo, MI, USA). This system provides preoperative planning, precise execution, and validation of the surgical outcomes [1,2,7].

Robotic-arm-assisted total knee arthroplasty (RATKA) employs a haptic-assisted robotic arm designed for bone preparation [1,7]. The range of motion of the cutting tool is constrained within a defined three-dimensional surgical plan derived from preoperative high-resolution (0.6 mm) computed tomography (CT) scans of individual patients [2,7]. This approach minimizes deviations in implant alignment, and existing research indicates that robotic-arm-assisted surgery yields superior precision in implant positioning [6,8]. It should be noted that RATKA represents the most recent application in the system in 2017, while the inaugural application of a robotic-arm-assisted technique took place in 2006 during a unicompartmental knee arthroplasty [9].

The purpose of this commentary is to present and categorize the limitations and problems that could occur during a RATKA surgery. Furthermore, ways of anticipating troubleshooting and proper solutions are discussed.

## 2. Troubleshooting and Limitations in RATKA

The limitations of the procedure may be divided into two major categories: (1) limitations related to the surgeon and (2) limitations related to the system.

Regarding the first category (limitations related to the surgeon), the prolonged duration of the procedure because of the extra time for the checkpoints, bone pin placement, and bone registration, as compared to a manual total knee arthroplasty (TKA), has been associated with the learning curve of the operation [10].

**Figure 1:**
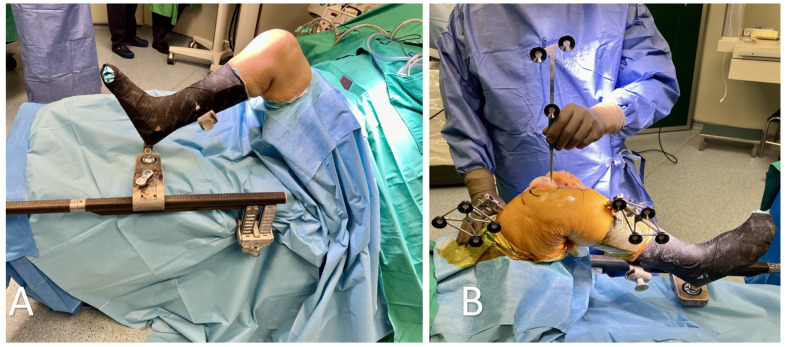
(**A**) Initial set-up with a stable placement of the lower limb in the leg holder. (**B**) The set-up of the robotic-arm-assisted total knee arthroplasty. The femoral and tibial arrays were placed, and registration with the probe was initiated.

The checkpoints and pin insertion are also surgical steps that, if not properly executed, may lead to troubleshooting later on. Loose implants may provide false data during the procedure, while additional incisions represent possible sites for wound complications. In particular, if the array becomes loose during the procedure, it may affect the accuracy of the procedure and change the visualization of the implants on the screen (Figure 1).

Probe registration may also be problematic since bone loss or cartilage defects may mess up the registration, leading to inaccurate surgical planning. The pressure applied to the probe during registration should be moderate, depending on the softness of the cartilage. Very deep penetration with the probe in order to register on a specific point may impair the level of accuracy. Moreover, during the mapping process, there may be some points that are not easily accessed due to the presence of osteophytes. Adequate mapping is required to proceed to the lateral and medial gap measurements and final bone-cut planning. Finally, another issue related to the surgeon is the soft-tissue injuries during bone cutting. Soft tissues, such as the patellar and popliteal tendons and the medial, as well as lateral ligaments, may be injured during this step, leading to an imbalance in knee extension and/or flexion, as well as disruption of the extensor mechanism. Table 1 summarizes the limitations that are surgeon-related, as well as some recommendations for avoiding them.

Regarding the second category (limitations related to the system), pin insertion and movement of the leg with the leg holder are difficult in patients with obesity and sort stature. Vibration during the operation (e.g., cutting) may lead to an intermittent function of the robotic-arm-assisted saw. The saw follows the planned cutting lines; therefore, the movement of the knee (due to vibrations in the cutting procedure) stops the procedure. Moreover, it should be noted that there are specifications regarding the operation room. The system weighs 460 kg and needs to be operated on a strictly flat surface. The system also needs to be linked to the power supply; otherwise, the batteries’ capacity may be reduced. The cost of pursuing the system is high, as is the cost of the service and updates of the software, and each operation also has a higher cost compared to the manual TKA due to the use of extra implants, including pins, leg holders, and reflectors with visa discs. The procedure is designed only for the Stryker prostheses, and the Mako Product Specialist (MPS) should always be present for (a) segmentation (planning), (b) verification of the registration, and (c) intraoperative optimization of the surgical plan. There are also some technical issues that may arise during the operation. The wireless connection between the components of the systems might be interrupted or lost, not enabling communication between the ongoing procedure and the execution of the surgical plan with the robotic-arm-assisted system. Furthermore, the robotic system has six joints with wires; if one of these wires breaks, the procedure cannot be completed with the use of the robotic system. If the wire in the saw is damaged, an exchange may occur in situ and the procedure may be completed. Table 2 highlights the limitations that are system-related, as well as some recommendations for avoiding them.

## 3. Discussion

RATKA represents a revolutionary advancement in orthopaedic surgery, offering precise patient-specific solutions for the management of degenerative knee joint diseases [7,11]. This groundbreaking approach combines the expertise of the surgeon with the precision and repeatability of robotic technology. By meticulously mapping the patient’s anatomy, the robotic-arm-assisted system optimizes implant placement and alignment, resulting in enhanced functional outcomes and improved implant longevity [1,5,12].

The reported outcomes of RATKA reveal improved accuracy in implant positioning, alignment, and restoration of joint biomechanics compared to conventional manual techniques [12,13,14]. Enhanced precision has led to reduced rates of malalignment and implant loosening, which may ultimately lead to better long-term functional outcomes and survivorship. Patients undergoing this approach reported improved postoperative pain relief, quicker recovery times, and increased patient satisfaction [2,7,12,13]. So far, the data from short and mid-term comparative prospective studies have revealed that patients undergoing RATKA, when compared to those undergoing manual TKA, have exhibited significantly better Knee Society and WOMAC scores [13,15,16,17,18]. No long-term data exist yet. Furthermore, the complication rates have been shown to be similar between RATKA and manual TKA. However, the cost-effectiveness and accessibility of this technology remain subjects of ongoing discussion. For robotics in knee surgery to have a significant impact, evidence showing enhanced implant durability and long-term patient satisfaction is necessary [19]. Such data are not yet available. If this occurs, we will witness a widespread adoption of robots and a swift evolution in technology.

Herby, we presented and categorized the limitations, as well as the pitfalls, during the use of the RATKA system. These issues can be classified into two main categories: surgeon- and system-related.

Regarding surgeon-related issues, the prolonged duration of the procedure is minimized as the learning curve is reached. Adequate training is required for all new systems and technologies. Studies have shown that the learning curve for RATKA ranges from 7 to 80 cases [20,21,22,23]. However, the majority of studies have revealed that the learning curve of RATKA in terms of surgical proficiency and surgeons’ confidence levels is relatively short, ranging from 7 to 11 cases [20,21]. Of course, this should be interpreted with caution since these studies included experienced surgeons with adequate knowledge of manual TKA.

Surgeons should also be careful during checkpoints and pin insertion to avoid loose implants that could provide false data intraoperatively.

Awareness of the position of these “extra” implants is of utmost importance throughout the procedure, especially during flexion and extension of the lower limb, as well as mapping and cutting. The femur and tibia require twp bone pins for each array clamp construct. In particular, 4 mm pins are used for the femur and 3.2 mm for the tibia. Regarding the femoral pins, the surgeon should flex the knee to more than 90 degrees in order to elongate the quadriceps muscles and then through an incision that is located more than 10 cm (or 4 fingers) proximal to the upper edge of the patella and about 30–35° medial of the midline, the pins should be inserted at about a 15 mm distance from one another. The Array Stabilizer is fully seated through both incisions so that the barrels are on the bone surface. Both cortices should be pierced in order to ensure stability. Positioning the bone pins about 30–35° inward from the midline helps prevent the risk of piercing the quadriceps muscle group, which could exert pressure and potentially displace the bone pins when the knee is bent. It should also be noted that to reduce the chances of femoral stress fractures after surgery, positioning the femur bone pins at the femur’s shaft should be avoided.

Regarding the tibial pins, through an incision that is located more than 10 cm (or 4 fingers) distal to the tibial tubercle and 1–1.5 cm medial to the tibial crest, the first 3.2 mm pin is placed, while the second is about 15 mm distal to the first one. Both cortices should also be pierced. To diminish the torque exerted on the bone during the array assembly, the array assembly is kept still while tightening the three thumb screws. If an array becomes loose and no cuts have been made, re-registration should be performed after ensuring firm fixation of the pins. If some cuts have already been performed, the accuracy of the following ones would be compromised; an attempt to restore the primary position is advisable

Regarding the checkpoints, caution is needed to ensure that they are placed as far as reasonably possible from the resection planes to avoid inadvertent resection during bone cuts.

**Figure 2:**
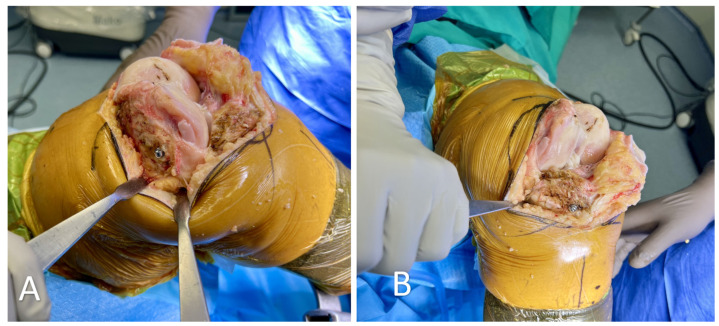
(**A**) Placement of the femoral checkpoint. Placement near the prominence of the medial femoral epicondyle, where the bone quality might be better, is advised. (**B**) Placement of the tibial checkpoint medially to the tubercle.

In cases where the femoral or tibial checkpoints become loose, accuracy may have been compromised. In order to avoid moving the array, the surgeon should avoid applying excessive force at checkpoints during validation, while it would be advisable to place the femoral checkpoint at the prominence of the medial femoral epicondyle where the bone quality might be better. Furthermore, the checkpoints should always be re-validated before using the blade saw (Figure 2).

**Figure 3:**
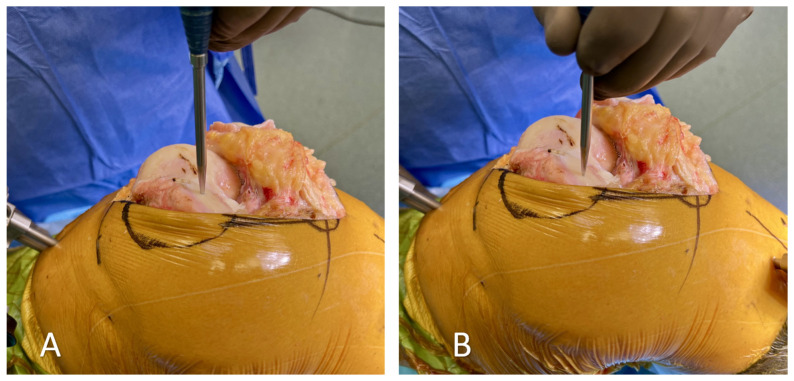
(**A**) “Wrong” way of registration with excessive force applied to the damaged cartilage. (**B**) “Right” way of registration with light touches of the probe.

Both the femur and tibia bone registration patterns contain 40 points (arranged in 10 groups of 4 points each). These points play a crucial role in establishing the anterior–posterior, medial–lateral, proximal–distal, and axial rotation (internal/external) alignment for each bone. Experience with the depth of each measurement during registration is also crucial. The adaption of the force applied with the probe in cases of cartilage defects holds significant importance during registration. This process has to be performed with light touches, so as not to penetrate the cartilage, which could be soft due to the degenerative disease. In the process of mapping, the surgeon should use the leg holder properly to allow flexion and extension, as well as rotation of the knee for reaching some difficult points; it should be noted that in some cases, a couple of mapping points may be skipped by re-verifying some extra registration points of the intact remaining cartilage (Figure 3).

Finally, the surgeon should always be aware of the soft tissues and the surgical plan for bone preparation. Although the system provides a cutting window that cannot be violated, information about the surrounding soft tissues is not included. We strongly recommend that the surgeon should not focus only on the system’s screen but also simultaneously watch the surgical field. The system can recognize and project only bone tissue. It is the surgeon’s responsibility to ensure that no damage occurs at the patellar tendon, as well as the medial–lateral ligaments, during this step. Moreover, it is important to take into account the balance of soft tissues that may alter the laxity after the bone cuts. Osteophytes may also affect the laxity; thus, medial and lateral gap measurements should be performed after the removal of all osteophytes in order to make an accurate surgical plan before the bone cuts.

**Figure 4:**
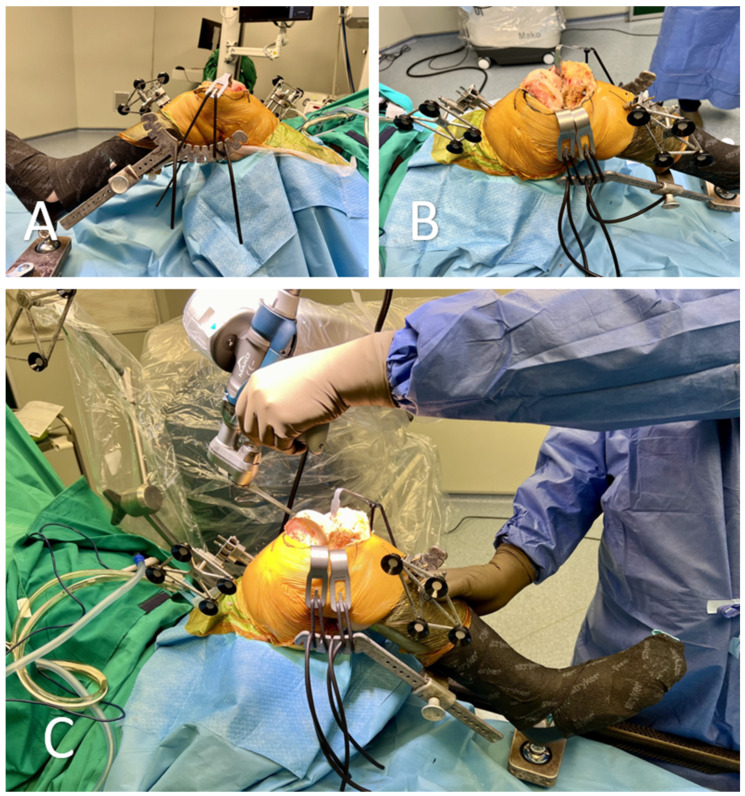
(**A**,**B**) Stable set-up during cutting with the leg holder and curved retractors. (**C**) The surgeon with one hand holds the robotic arm saw and with the other hand stabilizes the lower limb, avoiding interruption of the sawing process due to vibrations.

Regarding the system-related issues, it is important to use the leg holder in stable positions to minimize the interruption of the sawing process due to vibrations, while the surgeon can also stabilize the knee joint with the opposite hand (Figure 4).

In extremely rare cases where the wireless connection between the components of the system is interrupted, the connection can be bypassed using wired HDMI cables, and the operation may continue. Furthermore, in order to avoid issues with the six robotic joints and cable connections, a pre-operation check is always performed, including homing, camera accuracy, brake, and discrepancy check. A crucial barrier to the use of the robotic-arm-assisted system is the high cost, not only of the initial financial investment but also of the maintenance and the recurring cost of disposables and imaging [24,25]. Preoperative CT imaging also leads to radiation exposure of the patient, which has been estimated to be about 48 chest X-rays [26]. This aspect has not been sufficiently highlighted, but it should not be neglected. Hence, it is of utmost importance to be critical of the outcomes and cost-effectiveness of the robotic technology. For the moment, it seems that it would not be beneficial for low-volume centers to acquire this technology [24].

The present commentary provides valuable insights into the limitations and pitfalls of RATKA. Robotic-arm-assisted surgery is expanding, and it provides accurate execution of the surgical plan. Lowering robot costs and adopting image-free, non-device-specific robots may be a more “cost-effective” solution for the future expansion of this technology. Nevertheless, it should be noted that CT-based images and the combination of static, as well as dynamic information, offer valuable solutions in more challenging cases, such as cases with prior implants (internal fixations or anterior cruciate ligament reconstruction screws). It is rather difficult to suggest improvements regarding the checkpoints and arrays, which may lead to troubleshooting since these bone attachments remain very important for the accuracy of the system. However, some future developments may include more joints, such as the shoulder, revision, oncology, and trauma cases that require arthroplasty, and in the future, the system may not be a “close” one but it could include more implant designs and prostheses.

## 4. Conclusions

By utilizing the MAKO system, RATKA provides valuable intraoperative information to the surgeon while ensuring accurate execution of the surgical plan and improving implant positioning. To avoid pitfalls during surgery, adequate experience and training are required. Awareness of the “extra” implants during the whole operation and care of the soft tissues are of utmost importance. A good understanding of the system and its limitations may also play a crucial role in some cases.

## Figures and Tables

**Table 1:** Surgeon-related limitations and recommendations for avoiding them.

Limitations and Pitfalls (Surgeon-Related)	Recommendations and Mitigations
(1) Learning curve and duration of the procedure	Adequate training and experience
(2) Checkpoints and pin insertion	Ensure precise placement of pins and careful verification during the procedure. Avoid accidental movement.
(3) Probe registration	Adapt probe force for cartilage defects and perform registrations with gentle touches.
(4) Mapping	Utilize leg holders to access difficult points, and re-verify extra registration points
(5) Soft tissue injuries during cutting	Surgeon vigilance to prevent damage to surrounding soft tissues. Utilize the cutting window provided by the system, but do not rely on the system for the recognition of the soft tissues.

**Table 2:** System-related issues and guidance for minimizing them. J: Joint; OR: operating room.

Limitations and Pitfalls (System-Related)	Recommendations and Mitigations
(1) Patient’s short stature and obesity	Careful pin insertion and stabilization of the patient’s leg.
(2) Vibration during the operation	Ensure a stable operating environment to minimize vibration effects. Use the leg holder.
(3) OR floor requirements	Ensure a flat and sturdy OR floor for proper system function.
(4) Power supply and battery capacity	Regularly monitor and maintain the power supply and battery capacity.
(5) High cost	Consider the cost implications for purchasing, operations, and maintenance, especially for low-volume centers. Lowering costs and non-device-specific systems may lead to a wide expansion of robotics in the future.
(6) Mako Product Specialist presence	Ensure the availability of a specialist for critical system aspects.
(7) Stryker implants limitation	Only use Stryker implants with the system as it is a closed system. Future applications may include other prostheses making the system’s application wider.
(8) Wireless connection interruptions	Have backup wired connections in case of interruptions.
(9) Saw stopping due to cable issues	Maintain and replace cables and instruments as necessary.
(10) Robot arm joint issues (J1–J6)	Monitor and service robot arm joints to avoid interruptions during surgery. Perform pre-surgery checks.

## Data Availability

Not applicable.
